# Social Support and Well-Being of Chinese Special Education Teachers—An Emotional Labor Perspective

**DOI:** 10.3390/ijerph17186884

**Published:** 2020-09-21

**Authors:** Tung-Ju Wu, Lian-Yi Wang, Jia-Ying Gao, An-Pin Wei

**Affiliations:** 1 School of Management, Harbin Institute of Technology (HIT), Harbin 150001, China; tjwu@hit.edu.cn (T.-J.W.); lynnwly1@outlook.com (L.-Y.W.); JiayingGao@outlook.com (J.-Y.G.); 2 International School of Business and Finance, Sun Yat-sen University, Zhuhai 519082, China

**Keywords:** social support, deep acting, surface acting, well-being

## Abstract

Due to their high expectations, teachers often hide their real emotions and play a role that conforms to public expectations of educational work. Special education teachers face a group of students with physical and mental disabilities who have high heterogeneity and require individualized services every day. Using social support theory, this study discusses special education teachers’ emotional labor and well-being. A total of 439 special education teachers in China participated in this study. We collected data at two different time-points and verified the research hypotheses with hierarchical regression and structural equation modeling analysis. The research findings show the mediating role of emotional labor in social support and well-being. It is, therefore, suggested that schools should pay more attention to special education teachers’ mental health and provide them with regular guidance and support.

## 1. Introduction

Alongside the evolution of trends, social development, and education, teachers have transformed from pure instructors into general service providers [1,2,3,4]. Teachers assume the responsibilities of student cultivation and national development in their educational work. Society often measures teachers’ behavioral performance using high moral standards and expect them serve as examples to correctly lead students’ learning. Under these high expectations, teachers often hide their real emotions and conform to public expectations [1,2,4]. Special education teachers face a group of students with physical and mental disabilities who have high heterogeneity and require individualized attention every day. In addition to their daily teaching and marking homework, teachers have to provide individualized education plans, and deal with student issues according to each student’s individual differences [2]. By shouldering these responsibilities, special education teachers and their mental health statuses have received growing attention, especially with regards to their subjective well-being [4] as these issues not only affect quality of life but also directly affect the quality of special education. Prior studies have shown that the mental health levels of special education teachers is poor, and that the detection rate of mental health symptoms (such as poor well-being) is higher than that among other professionals [1,2,3].

Moreover, special education teachers have to assist in the promotion of the administrative businesses of schools. Such work severely tests them both mentally and physically, as their psychological stress exceeds that of regular teachers. Special education teachers more commonly present work stress and negative emotions [5] which will affect their work performance and willingness to work. Some higher correlations were found between the mental health levels of special education teachers and certain background variables, such as gender and teaching experience, as well as psychological variables, such as social support and teaching efficacy [1,5]. Special education teachers in special schools have to take particular care of intellectually disabled students in learning oral expression, social behaviors, physical functions, and life adaptation, due to the physical and mental characteristics of these students [3]. Therefore, such teachers must pay more attention to children’s physical and mental condition, quickly provide necessary assistance and fully present professional attitudes to offer children the greatest understanding. The pressure on special education teachers is thus very heavy [1]. Furthermore, teachers’ emotions are suppressed as they are generally presumed that they cannot randomly express negative emotions [4]. Without effective channels for release, teachers assume a greater emotional labor load [6,7,8]. Moreover, special education teachers require the cooperation and support of administrators. These cooperation processes, interpersonal interaction processes, and various types of support from others are the main ideas behind “social support” [2,4]. Accordingly, it is difficult for special education teachers to work as independently as regular teachers, as special education teachers require social support from administrators and regular teachers, related professional staff, and parents [1,4]. Special education teachers’ social support and emotional labor are, therefore, primary issues at present.

Special education teachers, particularly new special education teachers, express dissatisfaction and are thus particularly at risk for leaving the profession [2,3]. The findings of previous studies suggest several key factors related to the possible retention of teachers in special education: stress reduction, professional skill training, relationships with colleagues, and work autonomy [1,2]. Although many related studies have shown that there are close relationships between social support and mental health and between emotional reaction and mental health, there has been a lack of discussion on the relationship between the social support and mental health of special education teachers, as well as a lack of discussion on the relationships between special education teacher’s social support, emotional labor, and teaching well-being. In this study, we designed rigorous and detailed research to explore the relationships and mediating effects underpinning special education teachers’ social support, emotional labor, and teaching well-being and proposed the following hypothesis: that special education teacher’s emotional labor plays a mediating role in social support, thereby affecting special education teachers’ teaching well-being.

## 2. Research Model and Hypotheses

### 2.1. Social Support and Well-Being

Social support is a concept of social psychology. In social learning theory, individual behaviors refer to the interactions between individuals and the environment, which is a combination of the physical environment and the social environment. The idea of “society” generally refers to the social environment in which an individual resides and narrowly refers to the social situations with which an individual interacts in his or her daily life [9,10]. Rueger et al. [11] defined social support as the various patterns of support and assistance offered by other important individuals (e.g., family, friends, and colleagues) when an individual encounters pressure. These supports do not only share tasks but also provide extra economic, material, cognitive, and skill-based assistance to enhance an individual’s ability to adapt to the environment. From the perspective of dynamic processes, a social support system is a constant social aggregation process, which involves constantly contacting and interacting with others and using social networks to acquire feedback and affirmative support. From the perspective of personal perception, Bermejo-Toro et al. [8] regarded social support as an individual providing personal and family support through information exchange so that the individual perceives being loved and valuable and has a sense of belonging. French at al. [12] regarded social support as a type of social interpersonal exchange providing individuals with emotional, informational, and substantial assistance to satisfy psychological and material needs. Gray et al. [13] defined social support as other people’s unintentionally or intentionally helpful actions, including the interpersonal behaviors among diverse employees to enhance individual psychological or behavioral effectiveness. This factor can involve guidance, the provision of emotional support, collaboration, and the instruction of social capability structures.

Ju et al. [14] divided teachers’ social support into three dimensions for a questionnaire design. (1) Emotional support refers to resolving emotional concerns and providing support and encouragement through in various ways to induce special education teachers’ positive emotions. Special education teachers can receive care or assistance from the principal or administrators when encountering work stress via the provision of special education related policies, problem-solving programs, and respect for the special education teaching profession. Colleagues or professional staff can share their personal experiences or provide opinions when encountering problems and present active encouragement. Parents, spouses, children, or relatives can offer timely comfort or suggestions related to work frustration and/or provide work-related information [11]. (2) Informational support refers to the knowledge and experience offered through various types of support to assist teachers in solving related problems or dilemmas, e.g., analyzing problems or providing problem-solving strategies and sharing special-education-related professional information when special education teachers encounter problems or difficulties. (3) Substantial support refers to the substantial labor, materials, and time provided through various supports to solve special education teachers’ problems, such as the administrative units of schools offering specific assistance or resources, e.g., a good teaching environment, sufficient teaching equipment, and funds for special education teachers, or school colleagues’ cooperating to solve problems.

Jeong et al. [15] defined well-being as an individual’s overall attitudes towards his or her job. Employees with high well-being present positive attitudes, while those with low well-being manifest negative attitudes. Factors related to well-being include work being cognitively challenging, offering fair rewards, and the provision of a supportive work environment and colleagues. Maier et al. [16] understood job satisfaction as the way that employees perceive their job, as well as their individual attitudes towards various evaluations of work. Steele et al. [17] regarded job satisfaction as workers favoring jobs that consider job satisfaction, covering both environmental factors and individual factors. The former includes work characteristics, role variable, work-family conflicts, and treatment, while the latter includes personality, gender, age, and cultural difference [9,18,19]. Selvarajan et al. [20] highlighted job satisfaction as the pleasure acquired from work. Well-being is also called job satisfaction, where individuals consider their work process, content, and situation to agree with their intrinsic perceptions. Wu et al. [21] defined well-being as the degree that an individual and/or the majority of employees perceive(s) the well-being of their work, where the degree of well-being is related to salary, raises, work location, work time, type of work, promotion opportunities, interpersonal relationships, and management. Ilies et al. [22] suggested that well-being is determined by employees’ perceived work devotion and rewards, as employees compare their results with those of other members in the same organization; a more equal result will yield stronger feelings of well-being.

Most researchers measure well-being with the Satisfaction with Life Scale (SWLS) developed by Diener et al. [5]. This scale includes two dimensions of life satisfaction and positive emotions. Moreover, the Minnesota Satisfaction Questionnaire (MSQ, short form) developed by Lu [23] has been applied to the academic field for a long period of time, and the measurement of employees’ well-being offers good reliability and validity, where well-being is divided into “intrinsic satisfaction” and “extrinsic satisfaction”. (1) Intrinsic satisfaction refers to employees’ perceived well-being in terms of their value, responsibility, status, autonomy, self-esteem, and the sense of achievement induced by work. (2) Extrinsic satisfaction refers to employees’ perceived well-being related to salary, promotions, and work environment determined by work and interactions with colleagues.

Rueger et al. [11] highlighted the positive correlations between colleague and administrator support and well-being. Colleague support had strong positive effects on well-being, while administrator support showed indirect effects on well-being. French et al. [12] proposed significantly positive correlations between career mentors, coaches, work support, and well-being. Gustafsson et al. [13] indicated that stronger leader support and lower levels of role conflict, role ambiguity, and pressure can predict greater well-being and teacher retention. This study noted that administrator and family support provide higher well-being. Ju et al. [14] noted the higher predictability of the impacts of “supportive behavior” and “substantial behavior” in the organizational climate of schools on teachers’ well-being; moreover, the organizational climate of schools can effectively predict teachers’ well-being. Therefore, the following hypothesis was proposed in this study:

**Hypothesis 1** **(H1).** *Social support has a significant positive relationship with well-being.*

### 2.2. Social Support and Emotional Labor

Huang at al. [24] defined emotional labor as one’s personal control over their emotions to control the facial and physical expressions that can be seen by others. Moreover, emotional labor has exchange value and can be sold for wages. Chi and Grandey [25] regarded emotional labor as individual efforts at work or during interactions with people to adjust one’s emotion in consideration of the work [26,27]. Schaufeli et al. [28] noted that emotional labor is an individual emotion performed to conform to organizational or work expectations and requirements in the work field. Scherer et al. [29] defined emotional labor as an individual controlling their own emotions and applying language and physical movement to purposefully make customers perceive care, security, and a pleasant mood. Tucker et al. [30] regarded emotional labor as the workload resulting from an individual purposefully hiding or controlling his or her emotions to present the expected work impression. Wang [31] highlighted two ways in which workers act. Surface acting involves expressing one’s emotions with facial expressions, hand gestures, and tone, while deep acting aims to achieve the emotional performance requested by the organization. Geddes and Lindebaum [32] referred to surface acting as individual extrinsic behaviors through which a worker directly focuses his or her emotions, which is inconsistent with the worker’s intrinsic perceptions, such that the real emotions are not presented. Deep acting, on the other hand, refers to a worker’s emotions that directly focus on individual intrinsic perceptions and relate to extrinsic perceptions that conform to the emotional performance requested by the organization, such that the worker does not appear disorderly [27].

Hochschild [33] divided emotional labor into deep acting and surface acting: (1) Deep acting is regarded as being consistent with employees’ real perceptions and allows employees to truly “perform themselves”. Meanwhile, intrinsic emotions are adjusted to conform to the extrinsic emotional performance [34,35]. When real intrinsic negative emotions are involved, employees with deep acting attempt to make modifications to their performed emotions [18,33]. (2) Employees with surface acting are involved in the simulation of non-truly perceived emotions, attempting to adjust their extrinsic behaviors or inhibit their negative emotion without changing their intrinsic emotional performance, to be consistent with the organizational norms. Careful extrinsic verbal expression or the transmission of non-verbal information can present false emotions through facial expressions, posture, and tone [34,35].

Bermejo-Toro et al. [8] noted that acquiring affirmation, identification, or substantial needs and assistance from colleagues, friends, and family members through interpersonal interactions can allow one to receive social support while perceiving the relevant emphasis and concerns to properly manage the emotion, satisfy individual basic needs, and further achieve the performance requested by the organization. In the emotional labor model, Ju et al. [14] indicated that the environmental support of an organization can directly affect employees facing emotional labor. Here, work autonomy, supervisor suppers, and colleague support in the work environment are key factors. Geddes and Lindebaum [32] noted that the major effects of social support are to satisfy individual needs, enhance individual physical and mental health, and relieve the negative functions of pressure on an individual; thus, social support has positive effects on individuals. Accordingly, teachers need more support, identification, time, space, and resources to engage in emotional labor [33]. The administrators of schools can support teachers and provide positive emotional energy through informal organizations. Similarly, managers in profit-making institutions can provide emotional support through awards and praise, benign competitions, the assignment of challenging work, and the arrangement of assistance and training [8]. According to the above statements, we developed the following hypothesis:

**Hypothesis 2** **(H2).** *Social support presents significantly positive correlations with emotional labor.*

Wang [31] found significantly positive correlations between emotional labor load and well-being. Selvarajan et al. [20] observed positive correlations between kindergarten teachers’ emotional labor loads and well-being, where the higher the emotional labor load, the higher the well-being. Maier et al. [16] discovered remarkably positive correlations between teachers’ emotional labor load and well-being, as well as the predictability of the overall emotional labor load, deep emotional acting, and basic emotional expression on overall well-being. Zhang et al. [36] also determined that teachers with greater perceived emotional labor also perceived greater well-being. In addition to the positive correlation between special education teachers’ emotional labor and well-being, emotional labor is also predictably correlated to well-being [37]. Jeong et al. [15] determined the positive correlation between emotional labor and well-being, and emotional labor also correlated predictably to well-being. Mesmer-Magnus et al. [34] analyzed the relationship between emotional labor and well-being and determined notably positive correlations between deep acting and well-being. According to the above statements, the following hypothesis is posed:

**Hypothesis 3** **(H3).** *Social support will enhance teacher’s well-being through emotional labor.*

**Figure 1:**
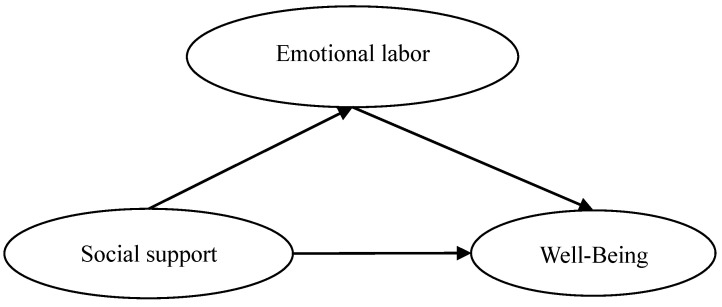
Theoretical model

The theoretical model of this study is shown in Figure 1.

## 3. Materials and Methods

### 3.1. Procedure

We contacted the special education teacher’s association in China to ask for their participation in our study. The data were collected at two time points. At Time 1, potential participants received an unsealed envelope that contained a cover letter explaining the purpose and procedure of this study and assured confidentiality, as well as a copy of the survey form. The participants were requested to provide informed consent and were assigned a unique code that allowed us to match the data collected later at Time 2. During the first round of data collection, 679 respondents completed the survey, including scales of social support and emotional labor. Respondents were requested to put the completed survey forms into the envelopes, seal them, and send them back to us. One month later, at Time 2, all 679 employees were contacted to complete the second survey to assess their well-being. Out of 679 special education teachers, 439 provided usable responses.

### 3.2. Participants

The 439 participants were from Beijing (27%), Shanghai (22%), Fujian Province (31%), and Guangdong Province (20%). Most of the participants were female (about 73.8%), the average age was 28.4, and the average job tenure was 5.4 years. All participants taught junior high school or senior high school. All had received a bachelor’s degree or above. More details are shown in Table 1.

### 3.3. Measurement

#### 3.3.1. Social Support

By revising the 40 questions in the Inventory of Social Support Behavior (ISSB) made by Barrera et al. [38], the social support scale in this study includes 10 questions under three dimensions of emotional support, informational support, and substantial support. It was measured using a 5-point Likert scale (1 = strongly disagree to 5 = strongly agree). To test the validity, the confirmatory factor analysis results provided reasonable suitability, component reliability, and average variance (χ^2^ = 814.72, degree of freedom (df) = 124, *p* < 0.05, Goodness of Fit Index (GFI) = 0.89, Adjusted Goodness of Fit Index (AGFI) = 0.86; Root Mean Square Error of Approximation (RMSEA) = 0.09 (<0.1); Standardized Root Mean Square Residual (SRMR) = 0.03 (<0.08), Normed Fit Index (NFI) = 0.97, Non-Normed Fit Index (NNFI) = 0.97, Comparative Fit Index (CFI) = 0.98, Incremental Fit Index (IFI) = 0.97, Relative Fit Index (RFI) = 0.97). The Cronbach’s *α* values of the sub-scales were 0.93, 0.91, and 0.92, and the *α* coefficient of the overall scale was 0.95.

#### 3.3.2. Emotional Labor

The operational definitions and measures of the dimensions in this study are based on those of Hochschild [33], including surface acting and deep acting in emotional labor. Surface acting refers to emotional labor workers who perform emotions that are nor real perceptions, and acting is presented verbally or non-verbally. Such emotional expressions generally follow organizational norms and are inconsistent with emotional labor workers’ intrinsic perceptions due to distinct intrinsic and extrinsic emotional expressions. Emotional labor workers attempt to change their real emotions to be consistent with the emotional expressions regulated by their organizations. This is deep acting. Since workers engage with consistent emotional rules related to perception, they can more naturally express the emotions expected by their organizations. Moreover, such workers do not easily exhibit affective disorder because of consistent emotional expression. This scale includes 11 questions and was measured using a 5-point Likert scale (1 = strongly disagree to 5 = strongly agree). Further confirming this validity, the confirmatory factor analysis results showed reasonable suitability, component reliability, and average variance (χ^2^ = 644.39, df = 117, *p* < 0.05, GFI = 0.91, AGFI = 0.87; RMSEA = 0.09 (<0.1); SRMR = 0.05 (<0.08); NFI = 0.96, NNFI = 0.96, CFI = 0.98, IFI = 0.98, RFI = 0.98). The Cronbach’s α values of the sub-scales were 0.93 and 0.89, and the *α* coefficient of the overall scale was 0.92.

#### 3.3.3. Well-Being

By integrating the Satisfaction with Life Scale (SWLS) developed by Diener et al. [5], the 13 total questions were measured using a 5-point Likert scale (1 = strongly disagree to 5 = strongly agree). Confirming the validity, the confirmatory factor analysis results revealed reasonable suitability, component reliability, and average variance (χ^2^ = 166.45, df = 113, *p* < 0.05, GFI = 0.94, AGFI = 0.92; RMSEA = 0.09 (<0.1); SRMR = 0.05 (<0.08); NFI = 0.97, NNFI = 0.96, CFI = 0.98, IFI = 0.98, RFI = 0.97). For the reliability, the Cronbach’s α of the sub-scales was 0.89 and 0.88, and the *α* coefficient of the overall scale was 0.91.

### 3.4. Methodology

All questionnaire data were input into a computer and analyzed with SPSS 21 (IBM, New York, NY, USA) and LISREL 8.72 (Scientific Software International, North Carolina) to determine the relations between social support, emotional labor, and well-being. During verification with structure equation modeling (SEM), maximum likelihood estimation (MLE) was used to estimate the parameters. Furthermore, according to Baron and Kenny’s [39] definition, the conditions to constitute mediating variables include the following. First, the predictor variable (A) can significantly predict the mediating variable (B). Second, the mediating variable presents remarkable predictability for a criterion variable (C). Third, a predictor variable also presents notable predictability for a criterion variable. Finally, the significant predictability of a predictor variable for a criterion variable is obviously reduced (even as low as zero (insignificant)) when the both predictor variable and the mediating variable are included in the regression model. The hypothesis of mediating variables is then supported. When the predictability of A to C is small but still remarkable, the predictability is mediated by B, i.e., partial mediation. When the predictability of A to C changes from significant to insignificant, the predictability is completely mediated by B (i.e., full mediation).

To test the mediation effect, Baron and Kenny [39] proposed to estimate three regression models. Testing the mediation effect with observed variables cannot exclude the measurement errors for which structure equation modeling (SEM) is utilized to test the mediation effect in this study. Furthermore, the SEM process allows researchers to estimate the errors of predictor variables, which conforms to the basic assumption of regression analysis. Moreover, when analyzing with latent variables, the measurement errors of the predictor variables and criterion variables are excluded in the SEM process, which can enhance the explanatory power of predictor variables for criterion variables. Finally, the variables considered in the regression analysis of the observed variables are limited can only prove the mediation effect. However, SEM can cover all variables in the analysis. Moreover, the casual context among variables better conforms to the variable relationships in the real world, thus providing better ecological validity.

### 3.5. Ethics Statement

Ethics approval was not required as per institutional guidelines and national laws and regulations because no unethical behaviors existed in this study. We only conducted paper–pencil testing and were exempt from further ethics board approval since our study did not involve human clinical trials or animal experiments. In the survey process, all participants were informed that participation was voluntary and assured that their responses would be only used for our study and kept strictly confidential. Therefore, only those who were willing to participate were recruited. To ensure confidentiality, the questionnaires completed during working hours were directly returned to the first author in sealed envelopes.

## 4. Results

### 4.1. Correlation Analysis

Table 2 shows the Pearson correlation coefficient matrix of the 439 participants in the seven observed indicators, as well as the means, standard deviations, and coefficients of skewness and kurtosis of each observed indicator. In the correlation coefficient matrix, most correlation coefficients achieved a significant standard of 0.05. In the test for the multivariate normality assumption, the chi-square value was 98.46 with *p* < 0.01. Although this result does not conform to the assumptions, the absolute values of the coefficients of skewness and kurtosis of all observed indicators are no larger than 2, with most below 1. Overall, these results conform to the basic requirements for maximum likelihood estimation.

To understand the predictions or explanations among variables, we adopted multiple regression to analyze our data with control variables and independent variables in the regression equation. For the prediction of surface acting with social support (shown in Table 3), the overall regression model (M2) test achieved significance of *F* = 34.87, *p* < 0.001, revealing the meaningfulness of the entire model.

### 4.2. Hierarchical Regression Analysis

The adjusted R^2^ = 0.35 shows that the dimensions of social support can explain 35% of the variation in surface acting. In collinearity diagnostics, the VIF is smaller than 10, so no serious collinearity exists. For the test of independent variables, the standardized regression coefficients of emotional support, informational support, and substantial support achieved significance and presented negative relations with surface acting. According to influence by numerical value, the variables are ordered as follows: emotional support (*β* = −0.28, *p* < 0.01), substantial support (*β* = −0.27, *p* < 0.01), and informational support (*β* = −0.22, *p* < 0.05). For the prediction of deep acting with social support, the overall regression model (M4) test achieved significance (*F* = 44.29, *p* < 0.001), showing the meaningfulness of the entire model. The adjusted R^2^ = 0.49 reveals that the dimensions of social support can explain 49% of the variation in surface acting. In Collinearity Diagnostics, the VIF smaller than 10 indicated no serious collinearity. In the test of independent variables, the standardized regression coefficients of emotional support, informational support, and substantial support achieved significance and presented positive relations with deep acting. According to influence by numerical value, the variables are ordered as follows: substantial support (*β* = 0.33, *p* < 0.01), emotional support (*β* = 0.31, *p* < 0.01), and informational support (*β* = 0.28, *p* < 0.01).

Furthermore, in the prediction of life satisfaction with social support, the overall regression model (M6) test achieves significance (*F* = 39.46, *p* < 0.001), showing the meaningfulness of the entire model. The adjusted R^2^ = 0.38 reveals that the dimensions of social support can explain 38% variation of life satisfaction. In collinearity diagnostics, the VIF was smaller than 10, showing that there is no serious collinearity. For the test of independent variables, the standardized regression coefficients of emotional support, substantial support, and informational support achieved significance. According to influence by numerical value, the variables are ordered as follows: emotional support (*β* = 0.32, *p* < 0.01), substantial support (*β* = 0.31, *p* < 0.01), and informational support (*β* = 0.29, *p* < 0.05), with positive predictability for life satisfaction. Finally, for the prediction of positive emotion with social support, the overall regression model (M9) test achieved significance (*F* = 31.27, *p* < 0.001), revealing the meaningfulness of the entire model. The adjusted R^2^ = 0.33 shows that the dimensions of social support can explain 33% of the variation in positive emotion. In collinearity diagnostics, the VIF was smaller than 10, showing that there is no serious collinearity. For the test of independent variables, the standardized regression coefficients of emotional support, substantial support, and informational support reached significance. According to influence by numerical value, the variables are ordered as follows: emotional support (*β* = 0.39, *p* < 0.01), informational support (*β* = 0.34, *p* < 0.05), and substantial support (*β* = 0.32, *p* < 0.01), with positive predictability for positive emotion.

According to the suggestion of Baron and Kenny [39], hierarchical regression was utilized for verifying the mediation effect. Three conditions are required. (1) Social support presented remarkable relations with well-being. (2) Social support showed notable relations with emotional labor. (3) When simultaneously using social support and emotional labor as the antecedent variables in the regression model, emotional labor presents significant relations with teacher well-being, but the predictability of social support decreases. As seen in M6 and M7 in Table 3, by adding the dimensions of emotional labor (surface acting and deep acting) into the prediction of life satisfaction with social support, the dimensions of social support, emotional support (*β* = 0.32 → *β* = 0.28, *p* < 0.01), informational support (*β* = 0.29 → *β* = 0.26, *p* < 0.01), and substantial support (*β* = 0.31, → *β* = 0.27, *p* < 0.01) are smaller but still show remarkable relations. Moreover, adding the dimensions of emotional labor (surface acting and deep acting) into the prediction of positive emotions with social support, the three dimensions of social support, emotional support (*β* = 0.39 → *β* = 0.27, *p* < 0.01), informational support (*β* = 0.34 → *β* = 0.29, *p* < 0.01), and substantial support (*β* = 0.32 → *β* = 0.24, *p* < 0.01) are smaller but still reveal notable relations. H3 is, therefore, partially supported. 

### 4.3. SEM Analysis

**Figure 2:**
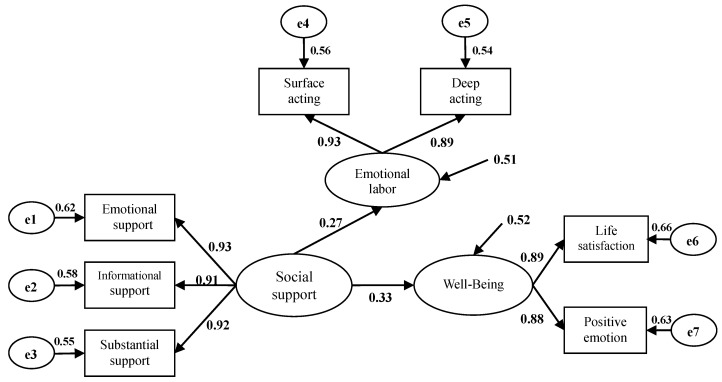
Mediating effect of emotional labor (step 1).

**Figure 3:**
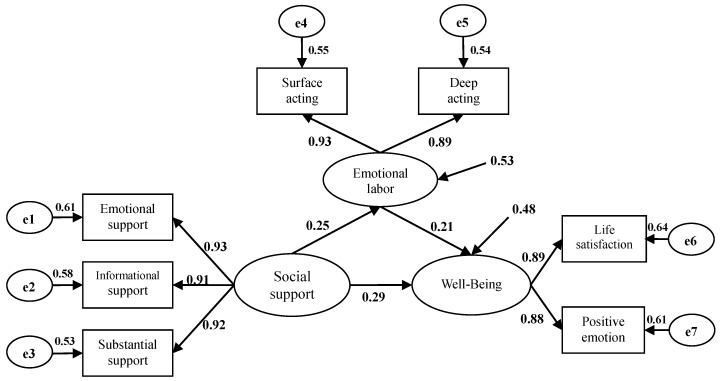
Mediating effect of emotional labor (step 2).

SEM is further used in this study to verify the mediation effect of emotional labor between social support and well-being. Figure 2 and Figure 3 and Table 4 show that predicting the mediating variable with social support in the emotional labor mediating path 1 (Figure 2) achieves significance (*γ*11 = 0.27, *p* < 0.01), and well-being also reaches significance (*γ*21 = 0.33, *p* < 0.01), agreeing with the assumptions of Baron and Kenny’s [39] regression Models I and II. In the emotional labor mediating path 2 (Figure 3), the prediction of well-being with emotional labor reaches significance (*β*21 = 0.21, *p* < 0.01), conforming to the assumptions of Baron and Kenny’s [39] regression Model III. Finally, the prediction of well-being with gratitude in Model II still reaches significance, but the effect is reduced. The above conditions conform to the requirement for SEM-mediating variables that emotional labor is the mediating variable of social support and well-being.

In summary, path 1 and path 2 present notable differences, while path 2 is more suitable for observing data. Moreover, both path 1 and path 2 conform to the requirement for SEM-mediating variables that the assumption of emotional labor is the mediating variable of social support and well-being. The mediation effect is discussed further in subsequent sections.

In the emotional labor mediating path 1 (Figure 2), the direct effect of social support on well-being reaches significance (*γ*21 = 0.33, *p* < 0.01). In mediating path 2 (Figure 3), the direct effect of social support on well-being is still significant (*γ*21 = 0.29, *p* < 0.01), but the effect is reduced, showing partial mediation. In other words, when social support and emotional labor are added to the model, the direct effect of social support on well-being is partially mediated by emotional labor. To further test the significance of the mediation effect, the Sobel test is applied. When the acquired z is larger than 1.96 or smaller than −1.96, the mediation effect achieves the significant standard of 0.05. The Sobel (*z* = 5.81, *p* < 0.05) result in this study shows that emotional labor indeed mediates the effect of social support on well-being, with a substantially meaningful mediation effect. Finally, the residual variation (ζ1) of emotional labor is 0.53. 

Figure 3 shows that the explanatory power of social support for emotional labor is 0.47, i.e., social support is able to explain 47% of the variation in emotional labor. Similarly, the residual variation (ζ2) of well-being is 0.48, indicating that the explanatory power of social support and emotional labor for well-being, is 0.52, i.e., social support and emotional labor are able to explain 52% of the variation in well-being.

## 5. Discussion

Some researchers have confirmed that social support can enhance the flexibility of an individual’s emotional responses to daily life [1,13,14], improve his/her mental symptoms effectively (such as anger, sadness, fear, and anxiety), and improve his/her quality of life. For the relations between social support and emotional labor, our research findings are similar to those of past research [7,40,41]. Past research indicated that social support will naturally enhance teachers’ emotional labor over time. Some research has indicated that social support can induce higher emotional labor [40,41]. Social support is a reaction to perceived interpersonal emotions. Individual perceptions of experience come from the mind inferring other people’s good intentions after receiving positive benefits to further induce affirmation and respect. In other words, when reflecting upon oneself as the beneficiary of others’ generosity, an individual will feel affirmed, respected, and valuable. This matches with social support providing individuals with feelings of being cared for, loved, respected, and valuable and engendering feelings of belonging. In this case, teachers would be willing to present their own identity; special education teachers would thus agree with their work and reduce their surface acting to promote their job and individual identity.

For the relationship between social support and well-being, our research findings are similar to those of past research [1,30,40]. Mathieu et al. [42] discovered that those with higher social support are likely to present higher positive emotions and feelings of well-being. For this reason, enhancing individual perceptions of social support will promote positive emotions and subjective well-being. Moreover, social support also has strong and positive relations with individual life satisfaction, energy, happiness, and subjective well-being [40]. Social support allows people to consciously perceive their importance to further perceive their well-being. It is very important to maintain an individual’s mental health by improving their level of social support, especially their understanding of social support [1,7,15]. In this study, mental health was also significantly related to optimism, which can affect the different factors of well-being: the highest correlation with self-esteem was found with the positive factors of mental health, and the highest correlation with depression was found with the negative factors of mental health. In other words, when special education teachers see their work affirmed by mass society, they become able to perceive and accept concerns or assistance from others. In addition to not taking the related benefits for granted, these teachers would also not take positive events in life for granted. This would help maintain individual happiness and well-being. Social support, therefore, could enhance physical and mental health and self-affirmation and promote individual self-value and psychological well-being. Good social support, therefore, can reinforce well-being.

In this study, the results of the regression analyses also demonstrated that certain factors of emotional labor can significantly predict various factors of well-being, showing that there is a certain causal relationship between emotional labor and well-being. However, social support could alleviate special education teacher’s well-being in emotional labor. This means that emotional labor plays a mediating role between the social support and well-being of special education teachers. In the emotional labor mediating Path 1 (Figure 2), the direct effect of social support on well-being reaches significance. In Path 2 (Figure 3), the direct effect of social support on well-being is still significant, but its effect notably reduced. This reveals that the direct effect of social support on well-being is partially mediated by emotional labor. According to the above, various emotional reactions in the practice of special education teaching could negatively moderate the effects of social support on well-being. By reducing emotional factors in the process of teaching, well-being could be alleviated to enhance the positive effects of social support on the well-being of special education teachers. Therefore, to improve special education teachers’ mental health levels, in addition to emotional management training, we should also seek to overcome and decrease negative emotions by improving teaching conditions, strengthening special education teachers’ training, and guiding colleagues to support special education teachers’ daily work.

### 5.1. Theoretical Contributions

Past research did not discuss the effects of social support on well-being through emotional labor. Accordingly, social support refers to an individual perceiving a positive value and attributing such value to others’ efforts. In addition to having an individual perceive being affirmed, respected, and valuable, and experiencing feelings of belonging, social support could help social behavior reinforce social network ties. On the other hand, the direct effect of emotional labor could enhance physical and mental health and self-affirmation, as well as promote individual self-value and psychological well-being. In this case, self-cognition could reinforce social support [7,40]. In other words, social support could directly and positively predict individual emotional labor, and good social support could also reinforce well-being [42] i.e., emotional labor is able to directly and positively predict individual well-being. Social support shows close relations with social bonds and social identity [42]. Special education teachers could thus perceive their social support, enhance their individual social benefits, and further promote their own subjective well-being. Inducing social identity through the expression of social support to further reinforce individual social networks and accelerate well-being relies on social support being able to enhance individual well-being through the reinforcement of emotional labor. The research results reveal the direct effect of social support on well-being and prove the indirect effects of emotional labor on well-being. Thus, emotional labor is the mediating variable of social support and well-being.

### 5.2. Implications for Practice

Social support has positive effects on well-being, indicating that special education schools should encourage their teachers to participate in society or organizational activities inside and outside school or even practice their plans through reward systems to enhance teachers’ participation motivation and reinforce social bonds and supports by establishing rich and diverse friend-level social networks to provide sufficient support when necessary. On the other hand, schools could hold seminars and workshops covering the theoretical and practical aspects of emotional and informational support for teachers or their family members to enhance their relatives’ ability to provide support. Thus, by reinforcing other people’s competence to provide required assistance and support in different situations could promote well-being.

Special education teachers’ psychological health education should be emphasized. In this case, we suggest integrating positive psychology theories into mentors’ study activities. The application of these theories to classroom teaching and life guidance can help teachers acquire positive psychological energy and/or release work stress and enhance their physical and mental health. Schools, therefore, should use mentors’ study time to reinforce special education teachers’ knowledge of positive psychology-related competence. Moreover, psychology-related courses could be established for teachers, and professional staff could be invited to enhance teachers’ counseling competence through an activity design of academic knowledge and actual applications in seminars and workshops. The aim is for teachers to become helpful friends of their students in school and deepen students’ gratitude intentions via an informal curriculum of teacher–student interactions to reinforce their positive psychological resources and enhance well-being.

### 5.3. Limitations and Future Research

Eventually, after integrating the relationships among social support, emotional labor, and well-being, the tests, comparisons, and analyses with structural equation modeling revealed the relevant influence paths and direct and indirect effects. This study provides a clear outline of the effects of gratitude on well-being for subsequent research. Thus, we have some suggestions for future research. We discovered that social support exerts direct effects on well-being and indirect effects through the mediation of emotional labor. In other words, there must be mediation from a third variable or more variables in the relationship between social support and well-being to have gratitude develop a larger effect. Future research could more deeply analyze other possible mediating variables (such as using upward and downward social comparisons and the cognition process of coding, retrieval, and extraction) to reduce the problems of surface or incorrect conclusions made by a third possible mediating variable when discussing dual-variable relationships. Moreover, under the trend of globalization, the value of self-identity cannot be denied. Thus, different demographic variables (e.g., gender, education attainment) could change the relationship between social support, emotional labor, and well-being. Moreover, would the difference between eastern and western culture enhance the distinct development and performance of social support? When a localization spirit is emphasized, would the factors of ethical norms, interactive relationships, and *renqing* and *mianzi* in Chinese society affect (i.e., enhance or hinder) the correlation between gratitude and well-being or generate a unique operational mechanism in the Chinese world to further construct a gratitude theory of Chinese culture? This factor is worthy of further discussion.

## 6. Conclusions

Drawing on affective events theory and positive psychology perspectives, this study examined the main effect of special education teachers’ social support on their well-being, as well as exploring the mediation effect of emotional labor. The findings deepened our understanding of the relationship between Chinese special education teachers’ social support and their well-being. For example, the direct effect of emotional labor could enhance physical and mental health and self-affirmation, as well as promote individual self-value and psychological well-being. Given the special education teachers are a specific party in the workforce and it is difficult for special education teachers to work as independently as regular teachers, this study can provide special education administrator with valuable information, helping them increase teachers’ well-being in the workplace.

## Figures and Tables

**Table 1:** The characteristics of the participants.

Variable	Category	*n*	%
Gender	Male	115	26.2%
	Female	324	73.8%
Location	Beijing	119	27%
	Shanghai	97	22%
	Fujian	136	31%
	Guangdong	87	20%
Age	28.4 (Standard Deviation = 5.82)		
Job Tenure	5.4 (Standard Deviation = 4.76)		
Teaching Grade Level	Junior high school	362	82.5%
	Senior high school	77	17.5%
Educational Degree	College or University	399	91%
	Graduate school and above	40	9%

**Table 2:** Means, standard deviations, correlations, and reliability among the study variables.

	M	S.D.	1	2	3	4	5	6	7
1. Emotional support	3.46	0.73	1						
2. Informational support	3.77	0.82	0.39 **	1					
3. Substantial support	3.18	0.67	0.31 **	0.37 **	1				
4. Surface acting	2.77	0.69	−0.28 **	−0.23 *	−0.22 *	1			
5. Deep acting	3.39	0.75	0.26 **	0.29 **	0.27 **	−0.22 *	1		
6. Life satisfaction	3.27	0.88	0.36 **	0.34 **	0.38 **	−0.27 **	0.24 *	1	
7. Positive emotion	3.59	0.47	0.39 **	0.35 **	0.39 **	0.22 *	0.27 **	0.37 **	1
skewness			−0.56	−0.29	−0.51	−0.27	−0.24	−0.38	−0.43
kurtosis			0.62	0.16	0.49	−0.02	−0.25	−0.14	0.33

*Note. n* = 439. * *p* < 0.05, ** *p* < 0.01, M refers to mean; S.D. refers to standard deviation.

**Table 3:** Hierarchical regression models: The mediating effect of emotional labor.

	Dependent Variable									
	Emotional Labor				Well-Being					
	Surface Acting		Deep Acting		Life Satisfaction			Positive Emotion		
	M1	M2	M3	M4	M5	M6	M7	M8	M9	M10
**Control variable**										
Gender	−0.18 *	−0.17 *	−0.18 *	−0.18 *	−0.21 *	−0.24 **	−0.24 **	−0.22 *	−0.21 *	−0.21 *
Age	0.25 **	0.25 **	0.24 **	0.24 **	0.19 *	0.18 *	0.18 *	0.18 *	0.17 *	0.17 *
Job tenure	0.29 **	0.23 **	0.28 **	0.26 **	0.14	0.15	0.15	0.17 *	0.15	0.15
Educational degree	−0.12	−0.13	−0.13	−0.3	−0.16 *	−0.13	−0.13	−0.18 *	−0.17 *	−0.17 *
**Dependent variable**										
Emotional Support		−0.28 **		0.31 **		0.32 **	0.28 **		0.39 **	0.27 **
Informational Support		−0.22 *		0.28 **		0.29 **	0.26 **		0.34 **	0.29 **
Substantial Support		−0.27 **		0.33 **		0.31 **	0.27 **		0.32 **	0.24 **
**Mediating variable**										
Surface Acting					−0.19 *		−0.17 *	−0.21 *		−0.18 *
Deep Acting					−0.24 **		−0.21 *	−0.23 *		−0.21 *
F values	21.18	34.87	19.71	44.29	20.46	39.46	56.32	18.48	31.27	47.65
AdjR^2^	0.18	0.35	0.21	0.49	0.17	0.38	0.52	0.15	0.33	0.46
ΔR^2^		0.17		0.27		0.21	0.14		0.18	0.13

*Note. n* = 439, * *p* < 0.05, ** *p* < 0.01.

**Table 4:** Test of direct, indirect, overall effect and significance.

	Dependent Variable			
	Emotional Labor		Well-Being	
	Standardized Effect	*t*-Value	Standardized Effect	*t*-Value
**Social Support**				
Direct effect	0.25	9.45 ***	0.29	10.27 ***
Indirect effect			0.04	3.36 ***
Overall effect	0.25	9.45 ***	0.33	12.58 ***
**Emotional Labor**				
Direct effect			0.21	4.28 ***
Indirect effect				
Overall effect			0.21	4.28 ***

*Note. n* = 439, *** *p* < 0.001.
